# Genetic Interactions of Progranulin Across the ALS-FTD Spectrum and Beyond

**DOI:** 10.17912/micropub.biology.000304

**Published:** 2023-12-21

**Authors:** James J. Doyle, J Alex Parker

**Affiliations:** 1 Division of Experimental Medicine, McGill University, Montreal, Quebec, Canada; 2 Metabolic Disorders and Complications, RI-MUHC, Montreal, McGill, Canada; 3 CRCHUM and Department of Neuroscience, University of Montreal, Montreal, Quebec, Canada

## Abstract

Progranulin (PGRN) is a growth factor in which mutations are one of the leading causes of frontotemporal dementia (FTD), and has been implicated in an assortment of neurodegenerative diseases. Conversely, higher levels of the protein have shown potential as a general neuronal protective factor. While examining its neuroprotective applications on a broader scale would be unfeasible in mammalian models, we turned to the nematode *C. elegans* to map the interactions of PGRN across multiple genetic models of neurodegenerative diseases. Our results indicate that while the overexpression of PGRN appears to be protective across all models tested, the loss of PGRN exacerbated the disease phenotypes of all but three of the models tested. Given the ease of genetic analysis in nematodes, we propose this model organism as an efficient tool to build a comprehensive map of PGRN’s genetic interactions.

**Figure 1. Genetic interactions of pgrn-1 in disease models:**
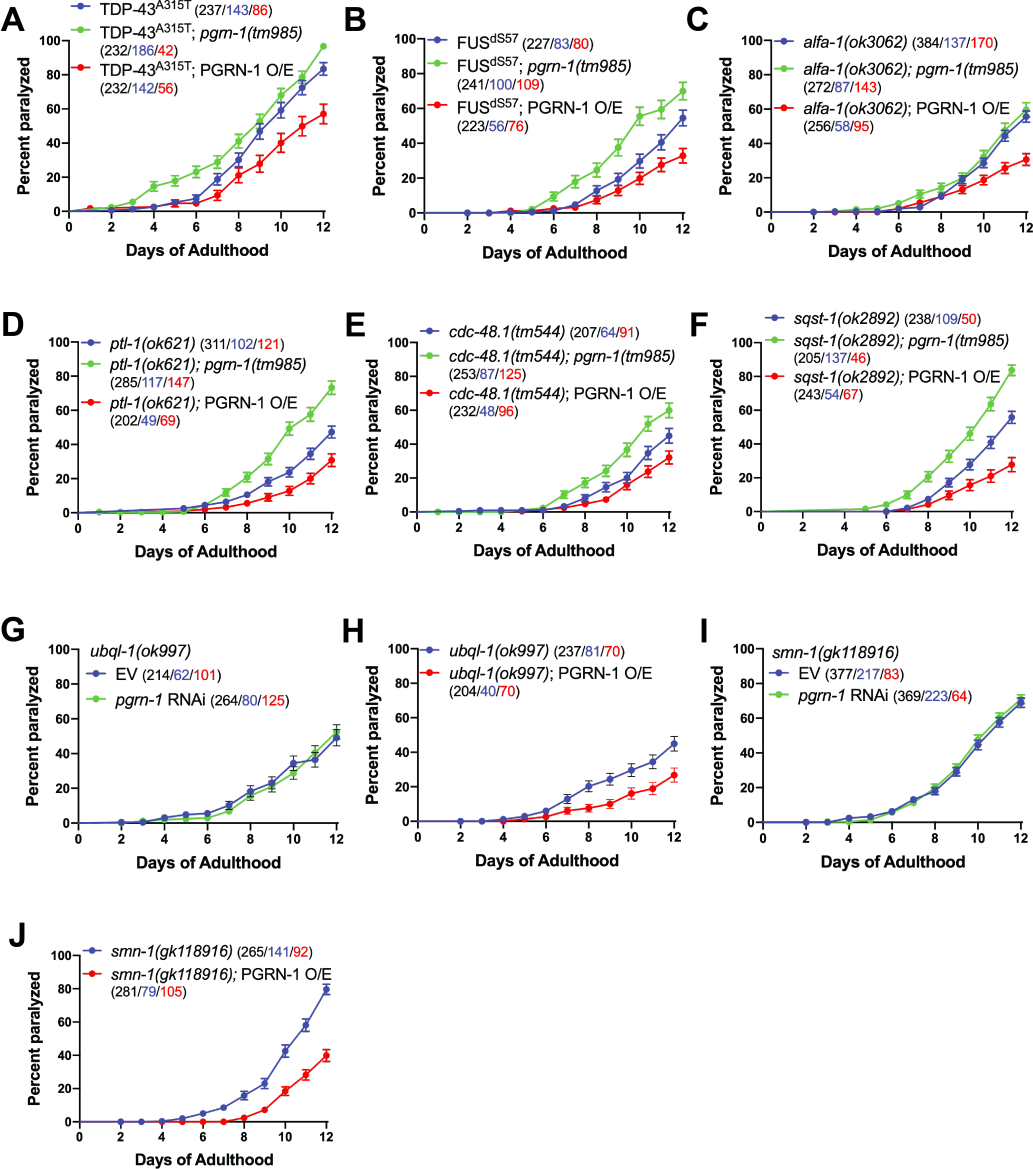
The genetic loss of *pgrn-1* exacerbates the paralysis phenotypes of TDP-43 ^A315T^ (A, *p* =0.0041) and FUS ^∆S57^ (B, *p* =0.0007) transgenic animals, and of *ptl-1(ok621)* (D, *p* <0.0001), *cdc-48.1(tm544)* (E, *p* =0.0016), and *sqst-1(ok2892)* (F, *p* <0.0001) mutant animals. Paralysis of *alfa-1(ok3062)* animals are not affected by the loss of *pgrn-1* (C, n.s.), and neither are *ubql-1(ok997)* (G, n.s.) and *smn-1(gk118916)* (I, n.s.) animals after depletion of *pgrn-1* by RNAi. RNAi experiments were conducted in *the uIs60 [unc-119p::YFP + unc-119p::sid-1]* background *.* (A-F, H, J) The overexpression of full-length, wild-type PGRN-1 ameliorates paralysis in all disease models tested (A, *p* =0.0003; B, *p* =0.0015; C, *p* <0.0001; D, *p* =0.0006; E, *p* =0.0232; F, *p* <0.0001; H, *p* =0.0011; J, *p* <0.0001). Data on graphs are presented as mean +/- SEM, gathered from multiple biological replicates. (PGRN-1 *O/E=PGRN-1 overexpressing strain; black numbers= total worm population; blue numbers= paralyzed animals; red numbers= censored animals)*

## Description

Progranulin (PGRN) is a highly-conserved protein and widely known to be one of the main genetic causes of frontotemporal dementia (FTD) (Baker et al. 2006; Cruts et al. 2006; Olney et al. 2017; van Swieten and Heutink 2008) , a primary form of early-onset dementia. While first identified as a secreted growth factor (Bandari and Bateman 1992; Bateman et al. 1990; Belcourt et al. 1993) , it has since been found to promote various neuropathologies when depleted or absent (Kao et al. 2017) and, more recently, as having important roles in brain aging (Rhinn and Abeliovich 2017) . PGRN, is known to be an efficient neuroprotective factor being able to delay or offset toxicity of numerous diseases across multiple animal or cellular models (Chitramuthu et al. 2010; Minami et al. 2014; Tauffenberger et al. 2013; Van Kampen et al. 2014) . The inverse is also true, wherein the loss of PGRN has been shown to exacerbate the severity of neurodegenerative diseases (Minami et al. 2014; Salazar et al. 2015) . As a result, many efforts are being put forth to develop PGRN into a general therapeutic for neuronal diseases. However, in order to better understand the potential applications of PGRN as a neurodegenerative therapeutic, its interactions with other neurodegenerative disease-causing genes must be examined in further detail. Furthermore, questions remain as to whether the protective effects of PGRN extend beyond other FTD, and the genetically-related ALS, genes (Ling et al. 2013) . However, performing such studies in mammals is costly and resource-intensive, so, other rapid models are required to perform these broad genetic interaction studies. In this study, we used the nematode *C. elegans* , to model PGRN’s genetic interactions across the ALS-FTD gene spectrum and beyond.

In order to map PGRN’s genetic interactions and verify its therapeutic potential against a specific genetic mutation, we crossed either *pgrn-1* -null animals, *pgrn-1(tm985)* (Kao et al, 2011; Doyle et al. 2021) *,* or multi-copy transgenic animals that overexpress wild-type *pgrn-1* under the control of its promoter (CF3778, denoted PGRN-1 O/E) (Kao et al. 2011) . We have previously shown that *pgrn-1(tm985)* animals display an adult-onset paralysis phenotype, which can be rescued by the overexpression of wild-type PGRN-1 (Doyle et al. 2021) , and we selected this phenotype to screen against. We sought to begin to map the interactions by looking at the most common disease-causing genes of the ALS-FTD spectrum. We crossed *C. elegans* *pgrn-1(tm985)* animals with transgenic nematode strains expressing ALS-linked, mutant, human TDP-43 and FUS, TDP-43 ^A315T^ and FUS ^∆S57^ (Vaccaro et al. 2012) . We observed that while the loss of *pgrn-1* exacerbated the paralysis phenotypes of each ALS model (Figs. 1A and 1B, respectively), the overexpression of PGRN-1 resulted in a suppression of paralysis. In order to test PGRN’s interactions with the most prevalent genetic cause of ALS and FTD, *C9orf72* , we turned to a genetic deletion in *alfa-1* , the nematode ortholog of *C9orf72* (Therrien et al. 2013) . We observed that, while the overexpression of PGRN-1 was able to supress *alfa-1* paralysis, the double mutant, *pgrn-1(tm985); alfa-1(ok3062)* did not have higher levels of paralysis as *alfa-1(ok3062)* mutants alone ( Fig. 1C ). We next sought to look at how PGRN interacts with MAPT whose ortholog in *C. elegans* is *ptl-1* . We found that the double *pgrn-1(tm985); ptl-1(ok621)* mutants displayed higher levels of paralysis than *ptl-1(ok621)* mutants alone, and that the overexpression of PGRN-1 resulted in lower paralysis levels ( Fig. 1D ).

We further sought to look at PGRN’s interactions with the less prevalent ALS-FTD causing genes, VCP, SQSTM1, and UBQLN2. When *pgrn-1* -null animals were crossed with *cdc-48.1* mutants, the worm ortholog of the human VCP, we observed an exacerbation of paralysis, whereas the overexpression resulted in a reduction of paralysis ( Fig. 1E ). We observed a similar pattern with *sqst-1(ok2892* ) animals, orthologous to the human SQSTM1 ( Fig. 1F ). For UBQLN2/ *ubql-1* , we could not cross *ubql-1(ok997)* animals with *pgrn-1(tm985)* animals since both genes are located on the same chromosome. We therefore turned to RNAi and fed *ubql-1(ok997)* animals RNAi against *pgrn-1* to deplete it, which we have previously shown induces a phenotype in wild-type animals (Doyle et al. 2021) . In doing so, we did not observe any exacerbation of mutant *ubql-1* paralysis ( Fig. 1G ). We were, however, able to cross PGRN-1 overexpressing animals with *ubql-1* mutants, which resulted in a reduction of *ubql-1* paralysis ( Fig. 1H ). Finally, looking beyond genes involved in the ALS-FTD spectrum, we looked at the effect of PGRN loss and overexpression on *smn-1* , the worm ortholog of *SMN1* known to cause spinal muscular atrophy (SMA) in humans. Much like *ubql-1* , *smn-1* is located on the same chromosome as *pgrn-1* so we had to turn to RNAi to deplete *pgrn-1* transcripts. We used here a newly-characterized nematode *smn-1* model, harboring the *gk118916* allele (Doyle et al. 2021) . In doing so, we observed that worms fed *pgrn-1* RNAi did not have any change in paralysis levels ( Fig. 1I ), whereas the overexpression of PGRN-1 resulted in a decrease of *smn-1* paralysis ( Fig. 1J ).

We observed that the loss of *C. elegans pgrn-1* resulted in an exacerbation of toxicity in all but three genetic backgrounds, *alfa-1* , *ubql-1* , and *smn-1* . In the case of *C9orf72/alfa-1* , this finding was of interest since previous studies have identified patients with both *GRN* and *C9orf72* mutations had pathology consistent with *C9orf72* mutations alone (Lashley et al. 2014) . However, another study has observed mixed pathology in patients carrying both *C9orf72* and *GRN* mutations therefore drawing uncertainty on this (van Blitterswijk et al. 2013) . Nonetheless, the *C9orf72* / *alfa-1* case is supported by recent findings suggesting that like PGRN, the C9ORF72 protein is involved in lysosomal function, and it has been hypothesized that its loss results in lysosomal function and trafficking defects which could be responsible for its pathology (Amick and Ferguson 2017; Amick et al. 2016; Shi et al. 2018) . Therefore, our finding is not surprising since it reinforces the suggestion that both *GRN* / *pgrn-1* and *C9orf72* / *alfa-1* may act through overlapping pathogenic mechanisms.

Unlike all the other genetic backgrounds we looked at, in the case of *ubql-1* and *smn-1* we could not introduce a *pgrn-1* -null allele through genetic crossing, so we turned to an RNAi approach to deplete *pgrn-1* transcript levels in the animals (Calixto et al. 2010; Fire et al. 1998; Kamath et al. 2001; Rocheleau 2012) . Therefore, as with the *alfa-1* results, our results suggest that *pgrn-1* contributes to pathology through similar pathways as *ubql-1* or *smn-1* . While there have been studies showing that UBQLN2 mutations can affect lysosomal-autophagic pathways, its primary role is in the ubiquitin-proteasome system (Renaud et al. 2019) . Furthermore, there is little evidence SMA pathology acts through lysosomal disruption (Chaytow et al. 2018) , so therefore, we believe that these results should be validated using genetically-edited mutants in *pgrn-1* in *ubql-1* and *smn-1* mutant backgrounds.

When looking at the effect that the overexpression of PGRN-1 had, we notice that it was able to reduce paralysis levels across all disease models tested, suggesting PGRN is, in fact, an effective and broad protective factor against neurodegenerative diseases; however, at this stage we cannot rule out that the tagged PGRN-1 is acting as a modifier of the phenotypes we are studying. Interestingly, it can rescue genetic disease models that do not have a known link to lysosomes, one of PGRN’s primary cellular functions, such as *smn-1* . This could suggest that one of PGRN’s mechanisms of action is on a pathway with broad-acting neuroprotective outcomes. Alternatively, PGRN has been shown to be a regulator of normal brain aging through its interaction with TMEM106b, a regulator of lysosomal function, whereby lower PGRN levels were associated with its accelerated aging, even in the absence of any other neuropathology (Rhinn and Abeliovich 2017) . Therefore, PGRN may promote neuronal health by maintaining normal lysosomal function.

Together, our results suggest that PGRN is, in fact, a broad-acting protective factor against a variety of neurodegenerative diseases. While we acknowledge this model system's limits to testing genetic interactions related to human diseases, our results provide a first stepping stone to elucidate the larger map of PGRN’s interactions.

## Methods

**Paralysis assays:** This assay quantifies the presence of the paralysis phenotype within a worm population. 25-30 L4 larval animals were placed onto NGM plates and scored daily starting the following day, at day 1 of adulthood. Worms were counted as paralyzed if they failed to move their body when prodded with a platinum worm pick. Worms were considered dead if they failed to respond to heat stimuli or if exhibited no pharyngeal pumping; dead worms were censored from statistical analyses. For each assay, a minimum of 200 animals were scored per genotype and per condition across 3 biological replicates. Animals were transferred to fresh plates every second day. Paralysis assays with RNAi were performed in the same way, and RNAi treatment was administered through feeding using standard protocols (Conte et al. 2015; Timmons et al. 2001) .

Statistical analyses: Paralysis curves were compared using Mantel-Cox tests calculated in GraphPad Prism software.

## Reagents

**Table:** 

Strain Name	Genotype	Short Name in Article	Source
XQ561	*pgrn-1(tm985)*	n/a	NBRP
CF3778	*pgrn-1(tm985); muIs213[pgrn-1p::pgrn-1::rfp]*	PGRN-1 O/E	
TU3311	*uIs60 [unc-119p::YFP + unc-119p::sid-1]*	n/a	CGC
XQ207	*xqIs133[unc- 47p::TDP-43[A315T];unc-119(+)]*	TDP-43 ^A315T^	
XQ209	*xqIs98[unc- 47p::FUS[delS57];unc-119(+)]*	FUS ^∆S57^	
XQ236	*alfa-1(ok3062)*	n/a	CGC
RB809	*ptl-1(ok621)*	n/a	CGC
RB1050	*ubql-1(ok997)*	n/a	CGC
FX544	*cdc-48.1(tm544)*	n/a	CGC
VC2196	*sqst-1(ok2892)*	n/a	CGC
XQ545	*smn-1(gk118916)*	n/a	MMP*

The strain resulting from the MMP (Million Mutation Project) was backcrossed to wild-type N2 animals 6 times. Other mutations were backcrossed 4 times.
